# A cross-sectional analysis based on GBD data: trends in the global, regional, and national burden of pelvic organ prolapse from 1990 to 2021

**DOI:** 10.3389/fpubh.2025.1555946

**Published:** 2025-05-09

**Authors:** Rui Wang, Wangshu Li, Jinliang Yang, Aziz ur Rehman Aziz, Chunfang Ha

**Affiliations:** ^1^General Hospital of Ningxia Medical University, Yinchuan, China; ^2^Key Laboratory for Early Diagnosis and Biotherapy of Malignant Tumors in Children and Women, Dalian Women and Children’s Medical Group, Dalian, Liaoning, China

**Keywords:** pelvic organ prolapse, global burden, incidence, mortality, disability adjusted life years, socio-demographic index

## Abstract

**Background:**

Pelvic organ prolapse (POP) is a prevalent condition affecting millions of women globally. Understanding its temporal trends and regional disparities is essential for effective public health interventions.

**Methods:**

A cross-sectional analysis was conducted using data from the Global Burden of Disease (GBD) study, examining trends in POP incidence, mortality, and disability-adjusted life years (DALYs) from 1990 to 2021. We analyzed the estimated annual percentage change (EAPC) and explored the association with the socio-demographic index (SDI).

**Results:**

From 1990 to 2021, global POP cases increased from approximately 8.4 million to 14.0 million, while the age-standardized incidence rate (ASIR) declined from 374.84 to 317.51 per 100,000 population (EAPC = −0.46). Deaths rose from 281 to 486, but the age-standardized death rate (ASDR) remained stable. DALYs increased from 232,432 to 389,358, with a decrease in age-standardized DALY rate (EAPC = −0.59). Low-SDI regions exhibited the highest ASIR, ASDR, and DALYs in 2021.

**Conclusion:**

The global burden of pelvic organ prolapse has increased in absolute numbers, although age-standardized rates have declined. Low-SDI regions continue to face the highest burden, highlighting the need for targeted healthcare interventions.

## Introduction

Pelvic organs prolapse (POP) is a common gynecological disorder that affects millions of women around the world. It is characterized by the abnormal descent of pelvic organs, primarily the bladder, uterus, rectum, and small intestine, into or beyond the vagina (1, 2). Estimates suggest that the prevalence of POP ranges from 3 to 6% in the general female population, with the incidence increasing to approximately 50% in parous women (3). This descent occurs due to weakened connective tissues and muscles that typically support these organs. Women with POP may experience various symptoms, including pelvic pressure, vaginal bulging, urinary and bowel dysfunction, and sexual dysfunction, especially in older populations (2). Accurately determining the prevalence of POP is challenging because definitions and diagnostic methods vary significantly across studies and clinical practices. Estimates suggest that the prevalence may be as high as 15% based solely on reported symptoms, but it can rise to 64.6% when evaluated through clinical examination (4, 5). This variability underscores the need for standardized diagnostic criteria to improve understanding and management of this condition. POP is characterized by high prevalence, the complexity of surgical treatments, the risks associated with different procedures, and the need for skilled professionals to perform surgeries effectively, all of which contribute to a significant healthcare burden (3). For instance, laparoscopic techniques may improve stress incontinence management but carry a higher risk of recurrence, which could lead to increased long-term treatment costs. This highlights the ongoing burden of POP on both patient health and healthcare resources (6).

Aging has long been identified as the main risk factor for the development of age-related diseases (7). The World Health Organization has set standards for assessing aging in humans, which clearly define phenomena associated with aging and categorize them as diseases (8). Worldwide, aging is often recognized as the most significant risk factor for pelvic organ prolapse. The prevalence of symptomatic POP among women aged 70–79 is notably high, with a reported rate of 18.6 per 1,000 (9). This condition remains a significant concern even in high-income nations. For instance, in the United States, the annual incidence of POP ranges from 1.5 to 1.8 per 1,000, with the highest rates found in women aged 60–69 (10). Given the aging demographic in the U.S., projections indicate a potential 50% increase in the number of women affected by POP by 2050 (1). Moreover, the projected increase in the number of women undergoing surgery for POP is anticipated to reach 48.1% by the year 2050, primarily due to the aging (11). POP remains prevalent among rural populations, with a significant number of women remaining untreated (12). Consequently, it is imperative to gather current and comprehensive evidence to inform the development of effective intervention strategies. Additionally, there is an urgent need for epidemiological research to investigate various aspects, including women’s perspectives on prolapse symptoms and the specific trends in incidence. The Global Burden of Disease (GBD) studies have been instrumental in providing detailed and comparable estimates concerning the epidemiology and burden of disease associated with POP. Therefore, this study presents findings from the GBD 2021 research, which details incidence rates, years of life lost (YLLs), years lived with disability, and disability-adjusted life years across 195 countries, with comparative analysis to data from 1990 and 2021. Additionally, it outlines trends in age-standardized prevalence and incidence rates over this period, providing valuable insights for the management of POP through informed policy adjustments, resource allocation, and health system planning.

## Method

### Data sources

This study conducted a cross-sectional analysis utilizing secondary data from the GBD 2021 study. The GBD 2021 study offers a comprehensive assessment of health impacts associated with 369 diseases and injuries, as well as 286 causes of death. Utilizing indicators such as incidence, mortality, disability-adjusted life years (DALYs), years lived with disability (YLDs), and YLLs, it encompasses data from 204 countries and territories. This evaluation employs the most recent epidemiological data and enhanced standardized methodologies to provide an extensive and up-to-date analysis (13). This information, overseen by the Institute for Health Metrics and Evaluation (IHME) at the University of Washington (14), USA, is available at: https://vizhub.healthdata.org/gbd-results. The author of this article is a member of the GBD Collaborator Network, and the paper was prepared in accordance with the GBD protocol. All personal and institutional data are anonymized and protected.

### Dataset selection

To investigate the burden of POP, we selected the GBD 2021 dataset for its comprehensive, up-to-date, and comparable epidemiological data, covering global, regional, and national levels from 1990 to 2021. This dataset was chosen due to its standardized analytical methods, which ensure reliability and consistency across diverse populations. Specifically, we extracted age-standardized rates (ASRs) and absolute counts of incidence, mortality, and DALYs, along with their temporal trends, directly from the GHDx.

### Inclusion and exclusion criteria

The study included all GBD 2021 data related to POP across 204 countries and territories. Inclusion criteria encompassed all available estimates of POP incidence, mortality, and DALYs. Exclusion criteria were: (1) countries or regions with incomplete or poor-quality data; (2) those not reporting incidence, mortality, or DALYs. However, due to the GBD’s advanced statistical modeling to estimate missing data, no countries or regions were fully excluded, ensuring a complete global analysis.

### Data collection and potential biases

POP data in GBD 2021 were derived from hospital discharge records, insurance claims, and a systematic literature review. Estimates were generated using the DisMod-MR 2.1 Bayesian meta-regression model, stratified by age, sex, year, and country (13). YLDs were calculated by multiplying case numbers by duration (until remission or death) and disability weights (DWs), which quantify non-fatal health impairment (15). YLLs were computed by multiplying deaths by age-, sex-, location-, and year-specific life expectancy, with DALYs obtained by summing YLDs and YLLs (15). Potential biases include: (1) Data source heterogeneity: Variations in data collection methods and quality across countries may affect estimate accuracy; (2) Diagnostic inconsistencies: Differences in POP diagnostic criteria across regions and studies; (3) Reporting delays: Lags in data reporting from some regions could introduce inaccuracies. To mitigate these, GBD employs advanced statistical models and correction techniques, enhancing data comparability and reliability.

Additionally, the relationship between disease burden and the Socio-Demographic Index (SDI) was examined. The SDI is a composite measure that reflects the average income per person, fertility rates, and educational attainment in each country or region. The 204 countries and regions were classified into five categories based on their SDI scores: low (SDI < 0.45), low-middle (SDI between 0.45 and 0.61), middle (SDI between 0.61 and 0.69), high-middle (SDI between 0.69 and 0.80), and high (SDI ≥ 0.80) (16).

### Confounding factors and missing data handling

Confounding was primarily addressed through age standardization, calculating age-standardized rates (ASRs) by applying age-specific rates to a standard population structure. This method eliminates the influence of age distribution differences across regions. Missing data in the GBD 2021 study were managed through several approaches: (1) interpolation using adjacent years or similar regions, (2) statistical modeling to predict missing values, and (3) the incorporation of uncertainty via 95% uncertainty intervals (UIs). These strategies ensure that estimates are complete and reliable for all regions, with UIs providing an indication of the data’s reliability and confidence.

### Statistical analysis

Between 1990 and 2021, a comprehensive analysis was conducted to assess the burden of female genital prolapse diseases. This study reported all estimates as absolute counts, age-specific rates, and age-standardized rates (ASRs) per 100,000 individuals to quantify the disease burden. In the GBD 2021 study, the ASR was calculated using the following formula:


$$\mathrm{ASR}=\frac{\Sigma_{i=1}^{A}\mathrm{ai}\;\mathrm{wi}}{\Sigma_{i=1}^{A}\mathrm{wi}}$$

where ai represents the age-specific rate in the i th age group, w stands for the number of individuals (or weight) in the corresponding age group from a chosen standard population, and a refers to the total number of age groups. To minimize the effects associated with variations in population age structure, all rates were age-standardized per 100,000 individuals. Instead of relying on the actual global population size for the year 2021, we applied the global standard population from the GBD 2021 study as the weighting factor.

Furthermore, linear regression analysis was employed to calculate the Estimated Annual Percentage Change (EAPC), which estimates the annual rate of change over a specified period and assists in evaluating trends in the Age-Standardized Rate (ASR). The linear regression model used was y=α+βx+ε , where x represents the calendar year, y corresponds to the natural logarithm of ASMR or ASDR, ε is the error term, and β represents the upward or downward trend in ASR.

The EAPC was calculated using the formula: *EAPC = 100 (exp(β) − 1)*. A 95% confidence interval (CI) was derived from the regression model to assess the significance of the trend. If both the EAPC and its 95% CI were above zero, the ASR was increasing. Conversely, if both were below zero, a decreasing trend was observed. If the 95% CI included zero, the ASR was regarded as stable. Statistical analyses and visualizations were performed using R software (version 4.3.2) and Microsoft Excel (2017). The R packages employed included “ggplot2,” “RColorBrewer,” “patchwork,” and “ggrepel.”

## Results

### Global burden of pelvic organ prolapse (POP) and trends

Globally, the incidence of pelvic organ prolapse (POP) has significantly increased over the study period, rising from 8,407,180.25 cases (95% UI: 7,070,339.38–10,061,267) in 1990 to 13,971,720.53 cases (95% UI: 11,651,598.21–16,614,082) in 2021. Concurrently, the age-standardized incidence rate (ASIR) of POP decreased from 374.84 to 317.51 per 100,000 population (95% UI). The estimated annual percentage change (EAPC) for incidence was −0.46 (95% CI: −0.5 to −0.42), indicating a decline in ASIR despite the rise in absolute case numbers. In terms of mortality, the number of deaths due to POP increased from 281.24 (95% UI: 218.05–372) in 1990 to 486.13 (95% UI: 352.43–704) in 2021. However, the ASDR remained stable, slightly decreasing from 0.01 to 0.01 per 100,000 population. Disability-adjusted life years (DALYs) attributable to POP also saw a rise from 232,431.53 (95% UI: 115,446.42–439,572) in 1990 to 389,357.96 (95% UI: 191,055–731,017) in 2021, while the age-standardized DALY rate decreased from 10.63 to 8.68. The EAPC for DALYs was −0.59 (95% CI: −0.63 to −0.56), reflecting an improvement in age-standardized measures despite the increasing absolute burden (Table 1).

**Table 1:** Age-standardized rates of incidence, deaths and disability-adjusted life years of POP in 2021 and their temporal trend from 1990 to 2021 at the global and regional levels.

Region	Deaths (95% UI, per 100,000 population)					DALYs (95% UI, per 100,000 population)					Incidence (95% UI, per 100,000 population)				
	All-age numbers		All-age numbers			All-age numbers		All-age numbers			All-age numbers		All-age numbers		
	1990	ASR 1990	2021	ASR 2021	EAPC 95%CI	1990	ASR 1990	2021	ASR 2021	EAPC 95%CI	1990	ASR 1990	2021	ASR 2021	EAPC 95%CI
Global	281.24 (372–218.05)	0.01 (0–0.01)	486.13 (704–352.43)	0.01 (0–0.01)	−1.35 (−1.55–1.14)	232431.53 (439,572–115446.42)	10.63 (20–5.26)	389,357.96 (731,017–191,055)	8.68 (16–4.26)	−0.59 (−0.63–0.56)	8407180.25 (10,061,267–7070339.38)	374.84 (449–314.29)	13,971,720 (16,614,082–11651598.21)	317 (378–267)	−0.46 (−0.5–0.42)
SDI region															
High SDI	87.21 (96–77.45)	0.01 (0–0.01)	67.06 (79–54.41)	0 (0–0)	−3.83 (−4.13–3.53)	48,951.49 (91,694–24041.44)	8.36 (16–4.09)	76,054.95 (144,659–37019.6)	7.82 (15–3.81)	−0.28 (−0.33–0.23)	1,664,153.14 (2,018,155–1,367,073.62)	300.13 (364–244.08)	2,619,299 (3,160,983–2168023.53)	296 (356–244)	−0.11 (−0.17–0.06)
High-middle SDI	28.68 (35–23.96)	0.01 (0–0)	44.22 (57–34.91)	0 (0–0)	−1.43 (−1.74–1.12)	41813.21 (79,597–20381.16)	7.54 (14–3.67)	67530.46 (127,220–32771.85)	6.52 (12–3.16)	−0.17 (−0.34–0)	1511689.42 (1,821,802–1237195.74)	279.05 (335–228.11)	2,429,035 (2,911,198–1976982.93)	247 (294–203)	−0.11 (−0.28–0.05)
Middle SDI	68.22 (85–57.41)	0.02 (0–0.01)	150.8 (227–105.25)	0.01 (0–0.01)	−1.45 (−1.67–1.23)	55254.1 (104,499–27617.74)	9.43 (18–4.7)	110702.78 (205,813–54217.91)	7.68 (14–3.81)	−0.62 (−0.66–0.57)	2105129.71 (2,515,534–1780995.29)	336.94 (400–284.52)	4,048,302 (4,829,315–3376929.65)	283 (335–239)	−0.49 (−0.54–0.45)
Low-middle SDI	71.86 (129–36.92)	0.03 (0–0.02)	169.66 (277–116.43)	0.03 (0–0.02)	−0.29 (−0.36–0.23)	62564.85 (120,205–31218.78)	17.49 (33–8.69)	93685.48 (179,646–47092.69)	11.36 (21–5.76)	−1.35 (−1.4–1.29)	2261051.83 (2,739,102–1878054.36)	572.16 (684–478.17)	3,322,801 (4,032,796–2724425.39)	382 (460–318)	−1.23 (−1.28–1.17)
Low SDI	24.93 (47–8.64)	0.03 (0–0.01)	53.94 (87–29.6)	0.03 (0–0.02)	0.31 (0.18–0.43)	23615.98 (45,617–11685.64)	17.77 (33–8.75)	41043.45 (78,906–20519.29)	13.07 (24–6.54)	−0.95 (−1.06−−0.85)	857024.5 (1,044,765–701361.62)	575.52 (691–472.32)	1,540,668 (1,882,647–1249681.32)	433 (524–355)	−0.85 (−0.94–0.76)
GBD region															
Andean Latin America	3.47 (5–2.15)	0.04 (0–0.02)	7.57 (11–5.43)	0.02 (0–0.02)	−1.75 (−1.96–1.53)	2230.57 (4359–1100.96)	18.85 (36–9.24)	4374.39 (8450–2134.43)	13.64 (26–6.66)	−0.99 (−1.06–0.93)	77908.62 (96,456–62819.16)	600.34 (735–484.73)	148,809 (184,461–119654.16)	452 (559–363)	−0.87 (−0.94–0.81)
Australasia	4.03 (5–3.29)	0.03 (0–0.02)	2.2 (3–1.44)	0.01 (0–0)	−4.2 (−5.4–2.98)	921.78 (1640–468.43)	7.55 (14–3.77)	1819.95 (3418–889.56)	7.2 (14–3.48)	0.01 (−0.16–0.17)	31292.82 (38,812–24681.91)	267.42 (330–209.4)	63,054 (78,473–49087.86)	271.55 (339–211.28)	0.17 (0.05–0.3)
Caribbean	4.5 (6–3.55)	0.03 (0–0.03)	8.74 (11–7.17)	0.03 (0–0.02)	−0.9 (−1.3–0.5)	2099.6 (4080–1026.95)	14.84 (29–7.33)	3148.07 (5925–1562.67)	11.39 (22–5.67)	−0.82 (−0.86–0.77)	71127.61 (89,534–57212.89)	478.91 (596–383.73)	100,801 (126,715–80159.15)	373 (471–297)	−0.75 (−0.79–0.71)
Central Asia	1.12 (2–0.84)	0 (0–0)	2.83 (4–2.1)	0.01 (0–0)	1.47 (1.29–1.66)	1820.55 (3427–836.5)	6.53 (12–3.01)	2719.23 (5175–1289.78)	5.61 (11–2.68)	0 (−0.24–0.24)	66388.28 (85,052–50634.85)	248.46 (318–188.34)	105,443 (137,050–80392.97)	214 (279–165)	0.01 (−0.22–0.24)
Central Europe	6 (7–5.15)	0.01 (0–0.01)	1.87 (2–1.47)	0 (0–0)	−6.87 (−7.77–5.97)	6538.35 (12,540–3050.79)	7.83 (15–3.66)	8454.23 (15,895–4056.2)	7.63 (14–3.63)	0.32 (−0.02–0.66)	231785.58 (294,816–182879.48)	298.62 (377–235.08)	284,421 (352,858–225966.16)	288 (361–232)	0.27 (−0.03–0.58)
Central Latin America	38.03 (41–35.2)	0.1 (0–0.09)	29.63 (34–25.68)	0.02 (0–0.02)	−5.5 (−5.87–5.13)	7394.54 (13,162–3927.47)	15.73 (28–8.35)	15663.82 (28,904–7623.75)	11.31 (21–5.51)	−1.18 (−1.3–1.05)	256814.49 (313,673–207445.99)	503.46 (611–404.62)	560,776 (682,827–448712.16)	400 (486–322)	−0.89 (−0.98–0.79)
Central Sub-Saharan Africa	3.05 (7–1.04)	0.04 (0–0.01)	8.33 (17–2.85)	0.04 (0–0.01)	0.39 (0.33–0.45)	1838.74 (3463–888.82)	13.3 (24–6.45)	3770.08 (7058–1874.07)	10.77 (20–5.49)	−0.59 (−0.68–0.51)	69405.94 (86,875–53951.28)	451.07 (564–353.35)	145,420 (184,949–112476.74)	365 (454–287)	−0.61 (−0.69–0.53)
East Asia	3.13 (10–1.39)	0 (0–0)	29.67 (43–8.3)	0 (0–0)	4.1 (3.06–5.15)	27021.97 (51,277–13187.01)	5.93 (11–2.87)	58616.76 (111,534–28402.38)	5.1 (10–2.47)	−0.32 (−0.47–0.17)	1088343.78 (1,300,015–898786.34)	233.34 (278–192.95)	2,252,958 (2,687,722–1837945.67)	203 (241–168)	−0.29 (−0.45–0.14)
Eastern Europe	10.95 (15–8.46)	0.01 (0–0)	16 (19–14.11)	0.01 (0–0.01)	−0.06 (−0.3–0.18)	13489.97 (25,617–6573.78)	7.73 (15–3.78)	15160.57 (28,604–7546.49)	7.5 (14–3.71)	0.73 (0.35–1.11)	470204.66 (576,006–379523.82)	293.79 (351–239.63)	519,282 (632,022–422405.88)	283 (338–234)	0.73 (0.35–1.11)
Eastern Sub-Saharan Africa	8.47 (17–2.78)	0.02 (0–0.01)	14.02 (23–5.36)	0.02 (0–0.01)	−0.94 (−1.02–0.85)	5634.78 (10,784–2818.9)	12.55 (23–6.18)	11304.97 (21,933–5686.58)	10.31 (19–5.11)	−0.55 (−0.61–0.49)	213787.4 (261,927–171021.19)	424.97 (514–342.69)	443,297 (546,265–354445.61)	355 (433–287)	−0.51 (−0.56–0.45)
High-income Asia Pacific	4.13 (5–3.43)	0 (0–0)	8.89 (11–6.3)	0 (0–0)	−3.42 (−5–1.81)	4372.95 (8314–2044.55)	3.85 (7–1.8)	7635.41 (14,302–3707.89)	3.37 (6–1.56)	−0.41 (−0.52–0.31)	175646.77 (215,841–140318.52)	156.65 (191–126.05)	279,297 (335,465–226013.98)	139 (170–112)	−0.4 (−0.49–0.3)
High-income North America	18.05 (20–15.39)	0.01 (0–0.01)	21.08 (25–16.87)	0 (0–0)	−1.6 (−1.76–1.44)	16262.17 (30,133–7909.32)	8.86 (17–4.32)	27848.63 (52,405–13746.3)	8.47 (16–4.12)	−0.63 (−0.8–0.47)	589410.49 (716,090–477513.26)	338.89 (408–276.7)	1,006,307 (1,217,199–826066.61)	336 (405–278.98)	−0.36 (−0.48–0.24)
North Africa and Middle East	2.21 (4–1.01)	0 (0–0)	2.26 (4–1.43)	0 (0–0)	−1.87 (−2.18–1.55)	13353.91 (26,621–6374.83)	13.89 (27–6.55)	29107.15 (58,295–13798.17)	10.91 (22–5.17)	−0.73 (−0.8–0.66)	499466.47 (624,596–393768.05)	475.8 (586–376.03)	1,086,261 (1,372,333–850260.76)	377 (466–297)	−0.7 (−0.76–0.65)
Oceania	0.12 (0–0.06)	0.01 (0–0.01)	0.38 (1–0.2)	0.01 (0–0.01)	0.4 (0.33–0.47)	110.29 (207–52.23)	7.04 (13–3.35)	270.75 (508–127.38)	6.64 (12–3.23)	−0.16 (−0.17–0.15)	4548.99 (5842–3475.58)	260.82 (327–202.21)	11,174 (14,755–8551.12)	244 (308–191)	−0.19 (−0.19–0.18)
South Asia	80.44 (154–34.79)	0.04 (0–0.02)	196.36 (331–123.65)	0.03 (0–0.02)	−0.66 (−0.78–0.54)	68176.26 (131,416–34287.41)	20.2 (38–10.06)	99541.58 (190,392–51040.76)	11.98 (23–6.15)	−1.64 (−1.71–1.56)	2460724.06 (2,984,545–2048868.99)	651.37 (781–545.1)	3,501,664 (4,213,757–2889246.55)	401 (479–335)	−1.47 (−1.54–1.39)
Southeast Asia	8.69 (15–4.73)	0.01 (0–0)	43.72 (84–20.14)	0.02 (0–0.01)	2.33 (2.1–2.57)	8940.26 (16,750–4301.51)	6.17 (12–2.97)	18929.56 (35,296–9251.91)	5.16 (10–2.55)	−0.49 (−0.52–0.45)	361193.59 (444,040–291236.51)	235.44 (284–190.83)	729,179 (888,737–582583.07)	194 (234–156)	−0.56 (−0.6–0.53)
Southern Latin America	1.61 (2–1.29)	0.01 (0–0.01)	1.39 (2–1.1)	0 (0–0)	−4.46 (−5.28–3.63)	2891.39 (5506–1354.67)	11.51 (22–5.41)	4365.87 (8252–2018.95)	9.72 (19–4.5)	−0.53 (−0.58–0.47)	97452.5 (121,010–76244.38)	394.35 (492–309.38)	149,340 (185,201–116781.97)	348 (435–269)	−0.43 (−0.49–0.36)
Southern Sub-Saharan Africa	3.84 (6–2.51)	0.03 (0–0.02)	8.35 (12–5.62)	0.03 (0–0.02)	0.29 (0.05–0.54)	1828.46 (3328–931.8)	11.18 (20–5.69)	3277.56 (6003–1658.47)	9.25 (17–4.68)	−0.56 (−0.6−−0.52)	67875.76 (81,520–56332.23)	390 (469–321.85)	121,019 (146,468–100,031)	326 (393–271)	−0.52 (−0.54–0.5)
Tropical Latin America	10.36 (12–9.14)	0.03 (0–0.02)	31.37 (36–26.58)	0.02 (0–0.02)	−0.37 (−0.57–0.17)	9831.77 (19,142–5036.43)	15.89 (31–8.29)	16,181.4 (30,339–8365.35)	11.7 (22–6.08)	−1.31 (−1.42–1.21)	3,48,188.21 (4,31,277–286333.35)	509 (623–426.66)	505,631 (6,02,804–433458.37)	375 (446–321)	−1.28 (−1.38–1.19)
Western Europe	66.44 (74–58.93)	0.02 (0–0.02)	39.31 (47–31.55)	0.01 (0–0)	−4.2 (−4.52–3.89)	29386.4 (55426–14552.02)	10.31 (20–5.11)	39547.84 (75471–18955.42)	10.13 (20–4.92)	0.07 (0.01–0.13)	911658.89 (1129719–730785.84)	348 (431–276.86)	1,262,562 (1,537,058–1,018,850.76)	363 (446–290)	0.2 (0.13–0.26)

### Regional differences and trends in POP burden by socio-demographic index

**Figure 1:**
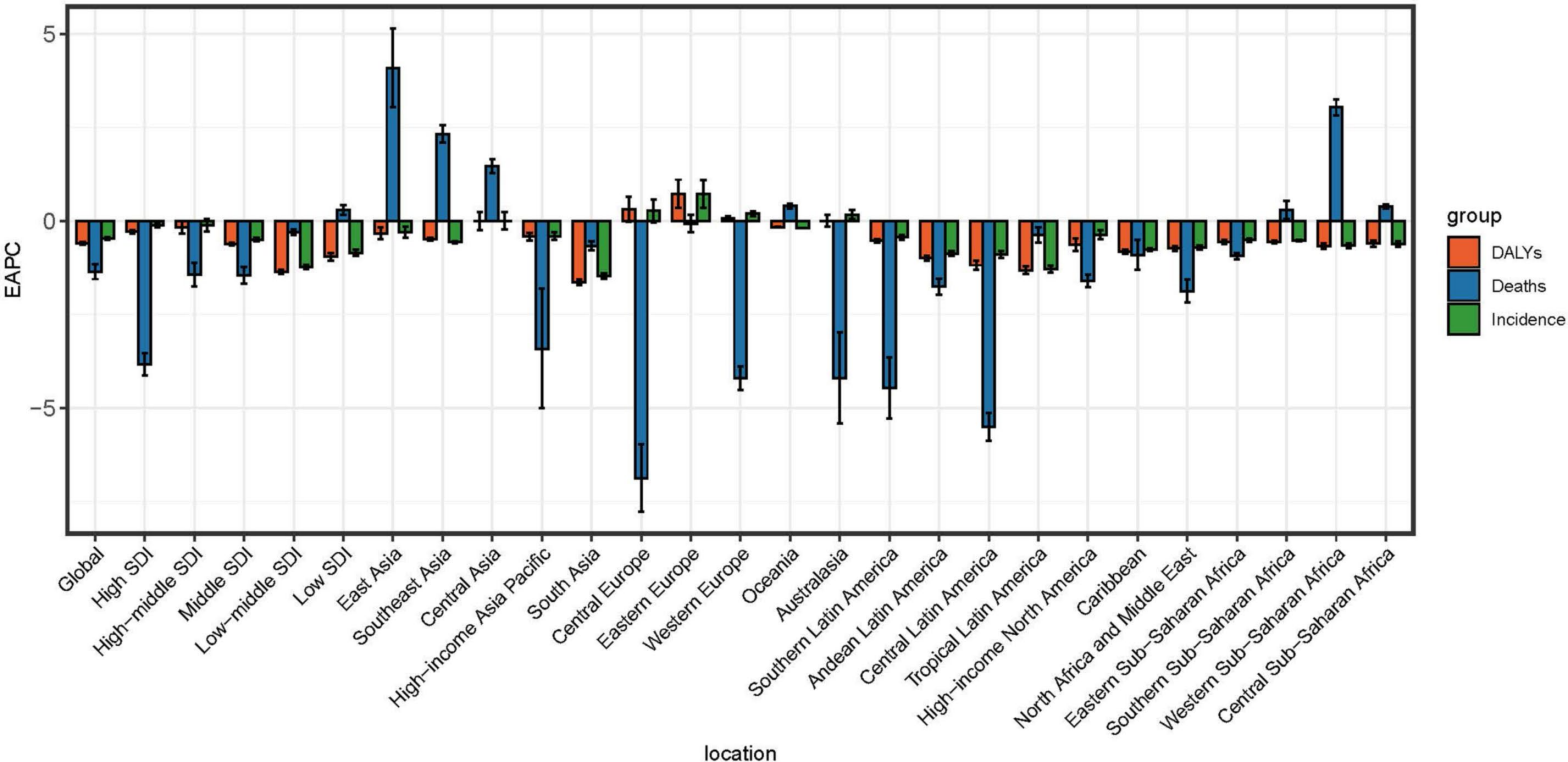
EAPC of age-standardized rates for incidence, mortality, and DALYs associated with female genital prolapse at global and regional levels. EAPC, Estimated annual percentage change.

As of 2021, the age-standardized rates for incidence, deaths, and DALYs of POP were highest in low-SDI regions. Across all SDI categories, the age-standardized incidence rates (ASIR) of POP exhibited a decreasing trend from 1990 to 2021, with low-middle SDI regions experiencing the most significant decline (EAPC = −1.23, 95% CI: −1.28 to −1.17). Regarding mortality, both high-SDI and high-middle SDI regions demonstrated a notable reduction in age-standardized death rates over the study period, indicating advancements in medical management and treatment. Similarly, the age-standardized DALY rates decreased across all SDI regions, with low-SDI regions showing the most substantial improvement (EAPC = −0.85, 95% CI: −0.94 to −0.76). These trends suggest that while the absolute numbers of POP-related incidents, deaths, and DALYs have risen globally, age-standardized metrics have improved across various socio-demographic contexts. Nevertheless, low-SDI regions continue to bear a disproportionately high burden of POP, underscoring the necessity for enhanced healthcare resources and targeted interventions in these areas (Table 1; Figure 1).

Table 1 Age standardized rates of incidence, deaths and disability adjusted life years of pelvic organ prolapse.

### Regional differences and trends by SDI

In 2021, low-SDI regions exhibited the highest age-standardized rates for POP incidence, deaths, and DALYs. Table 2 provides a summary of POP cases, ASIR, ASDR, and DALYs across SDI categories in 1990 and 2021, highlighting the distribution of the disease burden. Across all SDI regions, ASIR decreased from 1990 to 2021, with low-middle SDI regions showing the most significant decline (EAPC = −1.23, 95% CI: −1.28 to −1.17). For mortality, high-SDI and high-middle SDI regions demonstrated notable reductions in ASDR, while low-SDI regions experienced a slight increase (EAPC = 0.31, 95% CI: 0.18–0.43). Age-standardized DALY rates decreased across all SDI categories, with low-SDI regions showing the greatest improvement (EAPC = −0.85, 95% CI: −0.94 to −0.76). Despite these improvements, low-SDI regions continue to bear the highest burden of POP, underscoring the need for targeted interventions.

**Table 2:** Summary of POP cases, ASIR, ASDR, and DALYs by SDI category in 1990 and 2021.

SDI Category	Year	Cases (95% UI)	ASIR per 100,000 (95% UI)	Deaths (95% UI)	ASDR per 100,000 (95% UI)	DALYs (95% UI)	Age-Standardized DALY Rate per 100,000 (95% UI)
High SDI	1990	1,664,153 (1,367,074–2,018,155)	300.13 (244.08–364.00)	87 (77–96)	0.01 (0.01–0.01)	48,951 (24,041–91,694)	8.36 (4.09–16.00)
	2021	2,619,299 (2,168,024–3,160,983)	296.00 (244.00–356.00)	67 (54–79)	0.00 (0.00–0.00)	76,055 (37,020–144,659)	7.82 (3.81–15.00)
High-middle SDI	1990	1,511,689 (1,237,196–1,821,802)	279.05 (228.11–335.00)	29 (24–35)	0.01 (0.00–0.00)	41,813 (20,381–79,597)	7.54 (3.67–14.00)
	2021	2,429,035 (1,976,982–2,911,198)	247.00 (203.00–294.00)	44 (35–57)	0.00 (0.00–0.00)	67,530 (32,772–127,220)	6.52 (3.16–12.00)
Middle SDI	1990	2,105,130 (1,780,995–2,515,534)	336.94 (284.52–400.00)	68 (57–85)	0.02 (0.01–0.02)	55,254 (27,618–104,499)	9.43 (4.70–18.00)
	2021	4,048,302 (3,376,929–4,829,315)	283.00 (239.00–335.00)	151 (105–227)	0.01 (0.01–0.01)	110,703 (54,218–205,813)	7.68 (3.81–14.00)
Low-middle SDI	1990	2,261,052 (1,878,054–2,739,102)	572.16 (478.17–684.00)	72 (37–129)	0.03 (0.02–0.05)	62,565 (31,219–120,205)	17.49 (8.69–33.00)
	2021	3,322,801 (2,724,425–4,032,796)	382.00 (318.00–460.00)	170 (116–277)	0.03 (0.02–0.05)	93,685 (47,093–179,646)	11.36 (5.76–21.00)
Low SDI	1990	857,025 (701,362–1,044,765)	575.52 (472.32–691.00)	25 (9–47)	0.03 (0.01–0.06)	23,616 (11,686–45,617)	17.77 (8.75–33.00)
	2021	1,540,668 (1,249,681–1,882,647)	433.00 (355.00–524.00)	54 (30–87)	0.03 (0.02–0.05)	41,043 (20,519–78,906)	13.07 (6.54–24.00)

ASIR, Age-standardized incidence rate; ASDR, Age-standardized death rate; DALYs, Disability-adjusted life years; UI, Uncertainty interval.

### The regional burden of POP

In 2021, the highest ASIR of POP was observed in Andean Latin America (452.63, 95% UI: 559–363.61 per 100,000 population), South Asia (401.57, 95% UI: 479–335.15 per 100,000 population), and Tropical Latin America (375.45, 95% UI: 446–321.63 per 100,000 population). Similarly, the highest ASDR were found in Central Sub-Saharan Africa (0.04, 95% UI: 0–0.06 per 100,000 population), the Caribbean (0.03, 95% UI: 0–0.04 per 100,000 population) and South Asia (0.03, 95% UI: 0–0.04 per 100,000 population). The regions with the highest DALY rates were Andean Latin America (13.64, 95% UI: 26–6.66 per 100,000 population), South Asia (11.98, 95% UI: 23–6.15 per 100,000 population), and Tropical Latin America (11.7, 95% UI: 22–6.08 per 100,000 population).

In 2021, the ASR for deaths, DALYs, and incidence was the highest in the Low SDI region. Specifically, the Low SDI region had the highest ASDR (0.03 per 100,000 population), DALYs rate (13.07 per 100,000 population), and ASIR (433.51 per 100,000 population) globally. The Low SDI region also presented a slight increasing trend in age-standardized deaths rate (EAPC = 0.31, 95% CI: 0.18–0.43). For the age-standardized incidence rate, all SDI regions showed a decreasing trend, with the Low SDI region experiencing the fastest decline (EAPC = −0.85, 95% CI: −0.94 to −0.76). Similarly, the age-standardized DALY rate showed a decreasing trend across all SDI regions, with the Middle SDI region displaying the steepest reduction (EAPC = −0.62, 95% CI: −0.66 to −0.57) (Table 1). Globally, the age-standardized deaths rate and DALY rate have generally declined from 1990 to 2021. High SDI regions saw the most significant decline in deaths (EAPC = −3.83, 95% CI: −4.13 to −3.53), while Low SDI regions experienced a slight increase in the same metric. For DALYs, all SDI regions displayed a downward trend, reflecting improvements in health outcomes over the years (Table 1; Figure 1).

### The national burden of POP in 2021

**Figure 2:**
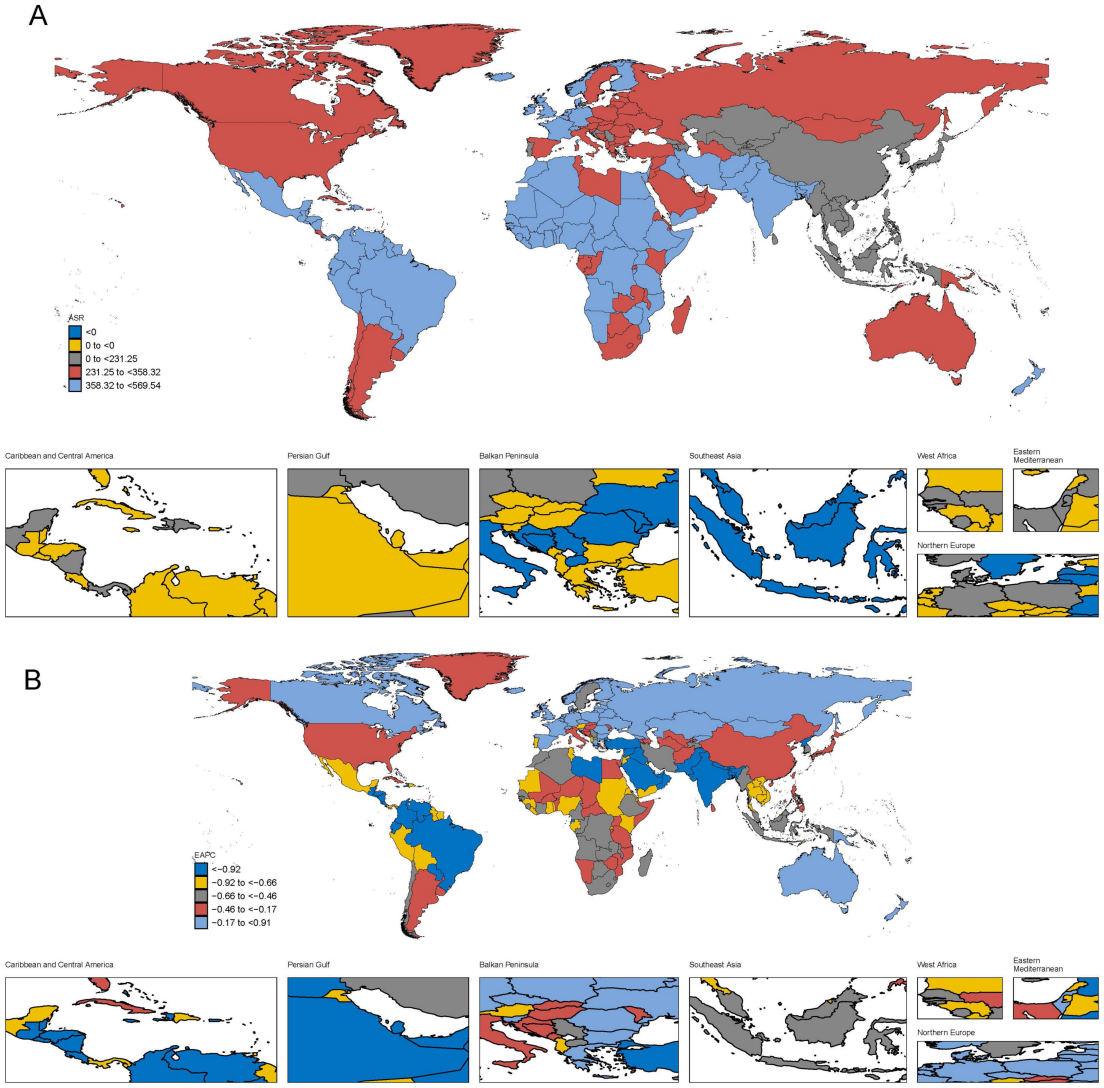
The global burden of POP in 204 countries or territories, in terms of incidence. **(A)** The ASIR of POP in 2021. **(B)** The estimated annual percentage change (EAPC) of ASIR of POP from 1990 to 2021.

In 2021, the highest ASIR was observed in India, with 2,584,373 cases (95% UI: 2,143,724–3,082,891), followed by China, United States, and Brazil (Table 1). These countries correspond to regions with high ASR levels in Figure 2A, where India and Pakistan are highlighted in light blue (ASR 358.32 to 569.54), representing the highest burden globally. Similarly, Egypt in Northern Africa also falls into this category, consistent with its notable contribution to the disease burden. Brazil, United States, and China, marked in dark red (ASR 231.25 to 358.32), represent regions with moderately high ASRs, reflecting substantial but comparatively lower burdens than the top-ranked countries. The geographic distribution in Figure 2A highlights significant regional disparities. South Asia, particularly India and Pakistan, and Northern Africa, represented by Egypt, emerge as critical hotspots requiring immediate public health interventions to address the high incidence rates and associated burden. Additionally, countries such as Brazil, United States, and China, although not in the highest ASR category, still contribute significantly to the global burden and require targeted healthcare strategies. Figure 2B focusing on EAPC (Estimated Annual Percentage Change), provides insights into trends in the burden of female genital prolapse. Regions marked in light blue (EAPC −0.17 to 0.91) indicate areas with slower or declining growth in burden, such as China and United States, while yellow regions (EAPC −0.92 to −0.66) show moderate declines, such as parts of Sub-Saharan Africa and Latin America. In contrast, dark red regions (EAPC −0.46 to −0.17), including India and parts of Southeast Asia, reveal ongoing challenges, as these regions experience slower declines or stagnation in disease burden reduction.

**Figure 3:**
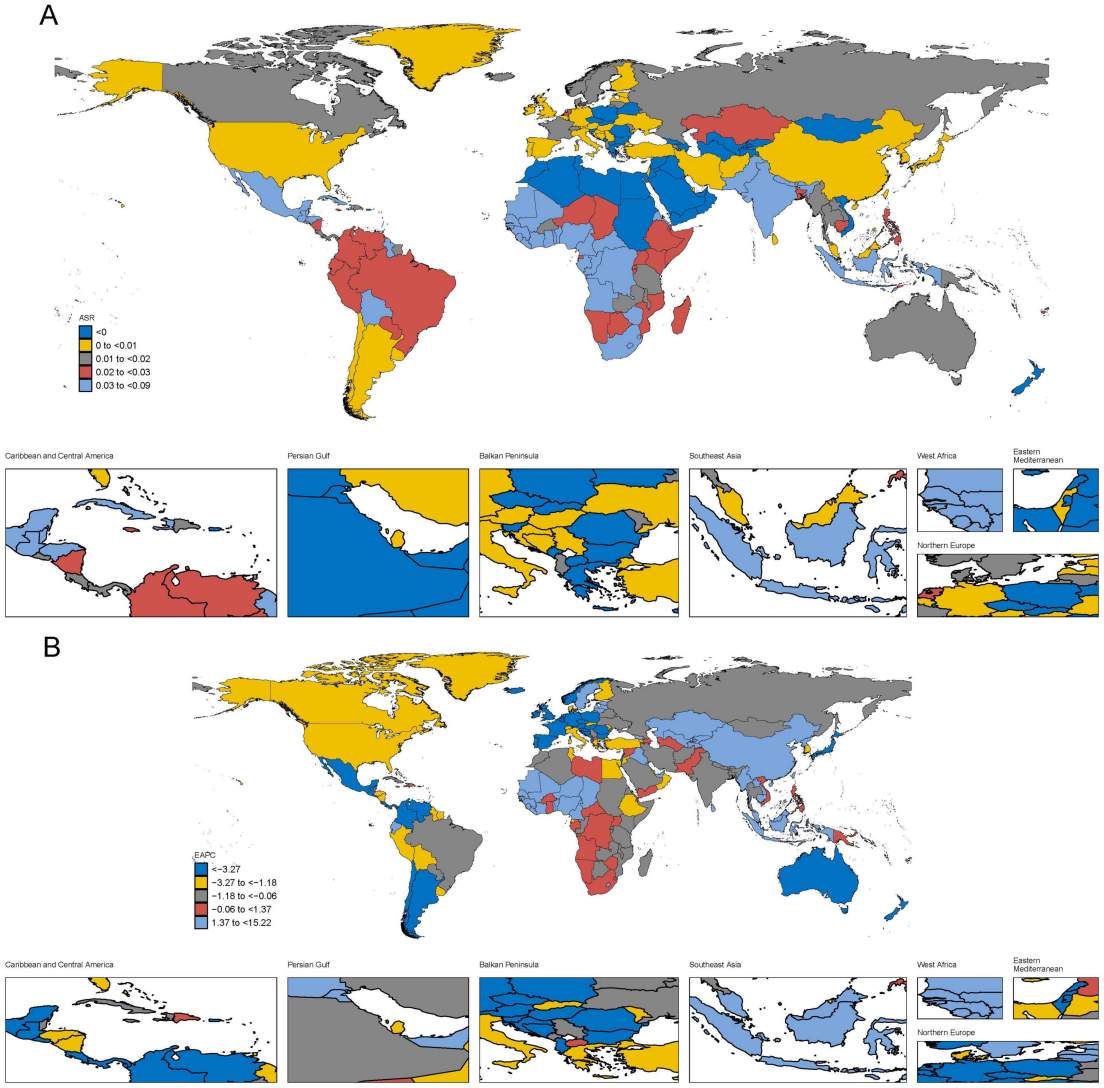
The global burden of POP in 204 countries or territories, in terms of deaths. **(A)** The ASDR of POP in 2021. **(B)** The EAPC of ASDR in POP from 1990 to 2021.

In 2021, the highest ASDR was observed in India, with 162.26 deaths (95% UI: 98.40–277.17), followed by Brazil (30.78, 95% UI: 26.07–35.46), China (27.84, 95% UI: 6.91–40.64), and Indonesia (24.98, 95% UI: 8.61–53.47). These countries correspond to areas marked in light blue and dark red in Figure 3A, where India and Pakistan demonstrate the highest burden globally. Brazil and China, shown in dark red, reflect substantial but lower contributions to the death burden compared to India. The geographic distribution in Figure 3A highlights that South Asia, particularly India and Pakistan, remains the epicenter of the highest death burden. Brazil, China, and Indonesia, although not at the highest level, still represent significant contributions to the global death rates due to female genital prolapse. From 1990 to 2021, the trends in ASDR across regions, depicted in Figure 3B, further illustrate a striking global disparity in the burden of POP-related deaths. South Asia, particularly India and Pakistan, experienced consistently high burdens but demonstrated modest reductions over time, with EAPC values near neutral or slightly negative. India showed a minimal decline, with an EAPC of −0.57%, while Pakistan also had a slight decline of −0.48%. Brazil, represented by yellow, showed a small reduction, with an EAPC of −1.04%. In contrast, significant reductions in ASDR, represented by dark blue, were observed in countries like Mexico and the United States, with EAPCs of −3.27% and −2.95%, respectively, reflecting effective healthcare interventions.

### Relationship between disease burden of POP and age

**Figure 4:**
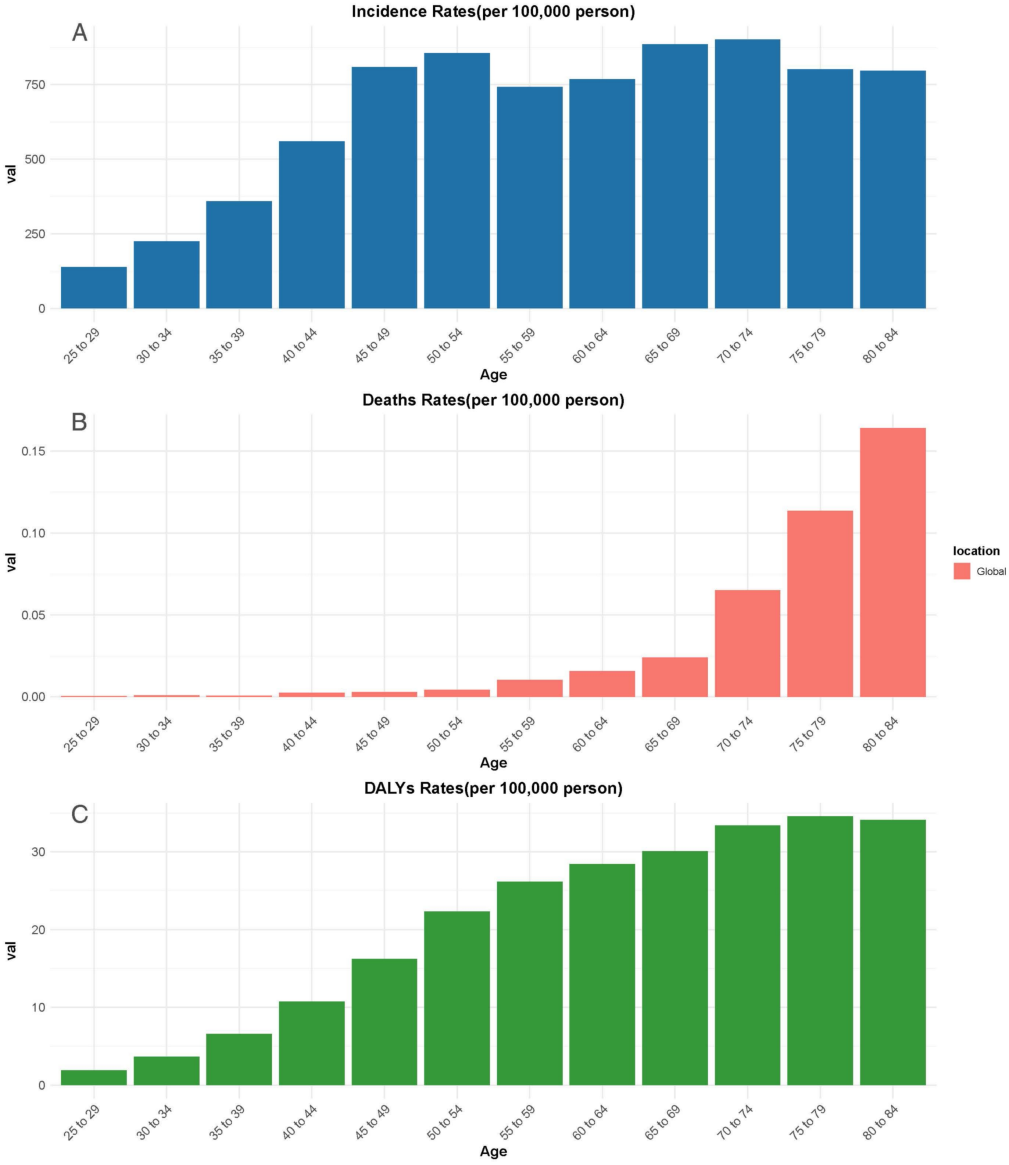
The incidence rate **(A)**, deaths rate **(B)**, and DALYs **(C)** of POP in different age groups globally. DALYs, Disability-adjusted life years.

Analyses of the incidence, deaths, and DALY rates of POP were conducted to observe the trends across different age groups (Figure 4). The incidence and DALY rates show a gradual increase with age, reaching their peaks in the 65–69 and 70–74 age groups, respectively. The incidence rate begins to rise from the 25–29 age group, with a noticeable surge after the age of 40. The highest incidence rates were observed in the 65–69 age group, before slightly decreasing in the older age groups.

The death rates, however, follow a different pattern. While remaining low in the younger age groups, there is a sharp increase after the age of 60, with the highest death rates occurring in the 80–84 age group. This suggests that as age increases, the risk of death due to POP becomes more pronounced, particularly in the older adults population.

Similarly, the DALY rates steadily rise with age, reaching their peak in the 70–74 age group, and remaining relatively high through to the 80–84 age group. This reflects the increasing burden of disability caused by POP as individuals grow older, contributing significantly to the overall disease burden in older populations.

### Association between POP burden and SDI level

**Figure 5:**
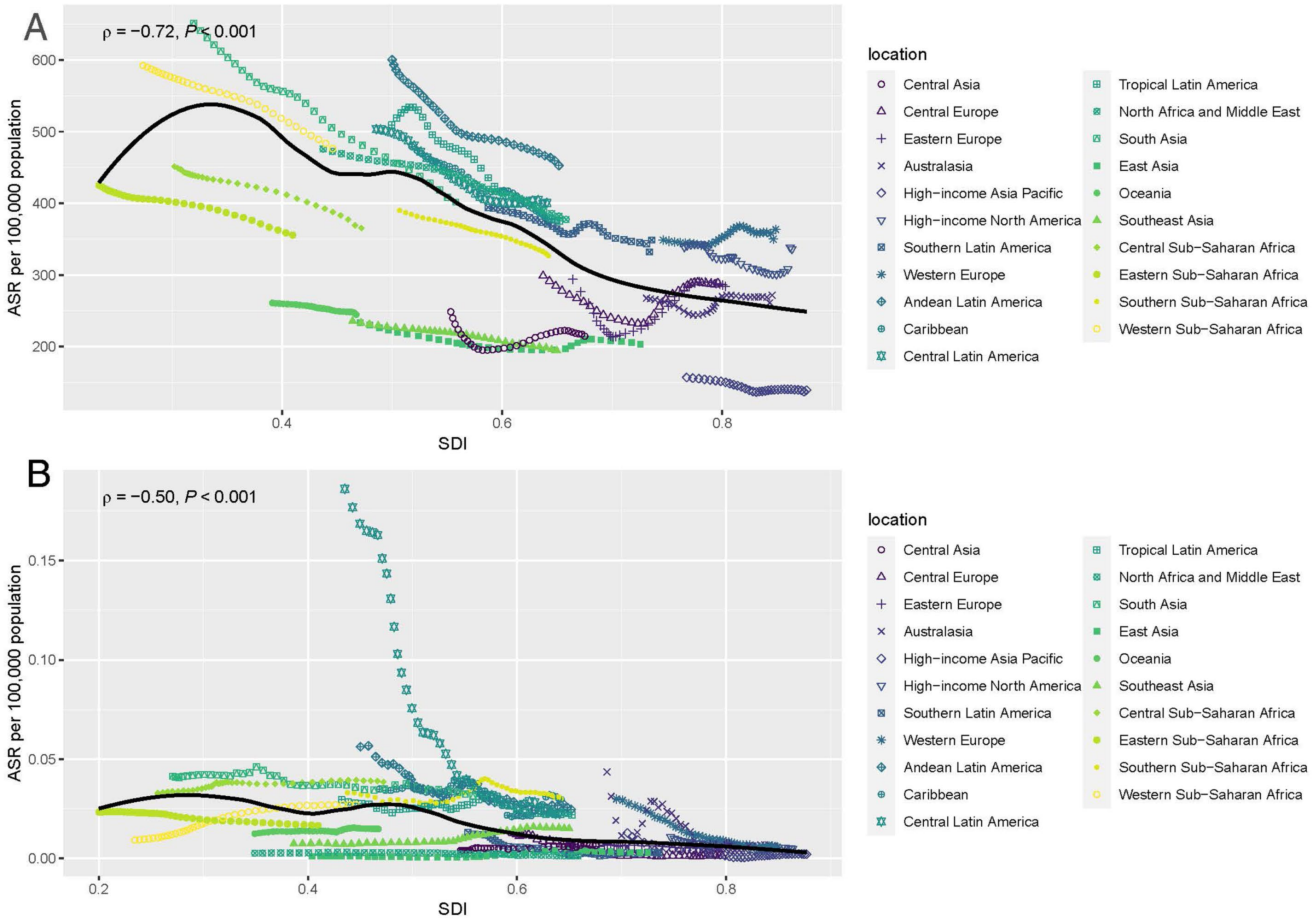
Age-standardized rates of incidence **(A)** and deaths **(B)** of POP by SDI from 1990 to 2019, for 21 geographic regions against their SDIs. SDI, Socio-demographic index.

In Figure 5, the association between the estimated burden of POP and the SDI level across 21 regions from 1990 to 2021 is displayed. As shown in Figure 5A, the estimated burden of POP incidence increases with SDI, reaching a peak at around 0.6 SDI, after which the incidence starts to decline with higher SDI levels. A similar trend can be observed in Figure 5B, where the estimated burden of POP deaths increases with SDI and peaks at approximately 0.5 SDI. After this point, the death rate begins to decrease as the SDI level continues to rise. This suggests that while POP burden is initially higher in regions with moderate SDI levels, the burden tends to decline as regions progress to higher SDI levels, likely due to better healthcare infrastructure and preventive measures.

### Temporal trends in the burden of POP from 1990 to 2040

**Figure 6:**
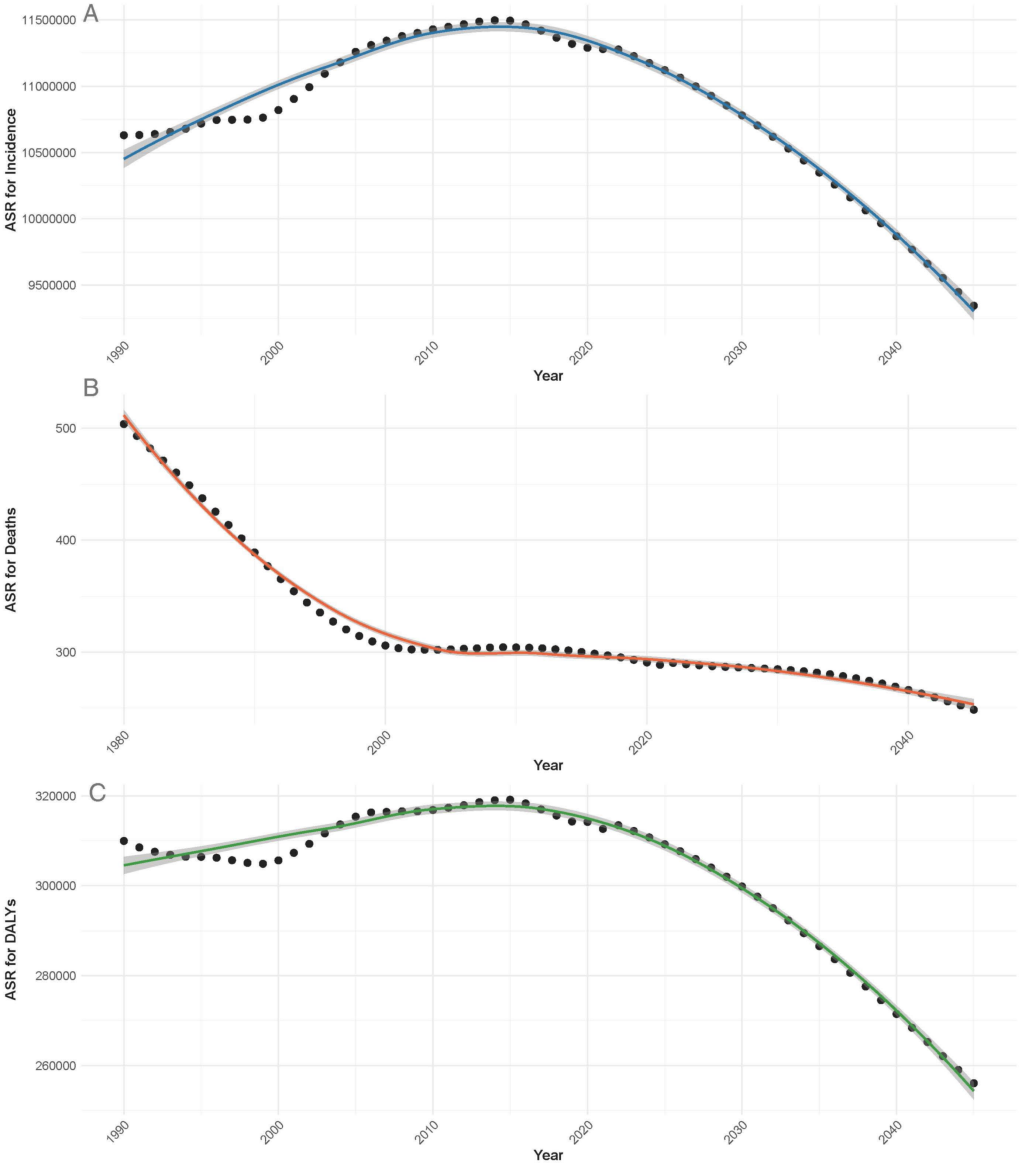
Temporal trends in the ASR for incidence **(A)**, deaths **(B)**, and DALYs **(C)** of POP from 1990 to 2040.

In Figure 6, the temporal trends for the incidence, deaths, and DALYs associated with POP from 1990 to 2040 are illustrated. As depicted in Figure 6A, the ASR for incidence shows a steady increase from 1990, reaching its peak around 2010, followed by a gradual decline projected to continue until 2040. This suggests that while the incidence of POP initially rose, improvements in prevention and healthcare are likely contributing to the expected future decline.

In Figure 6B, the ASR for deaths demonstrates a continuous downward trend since 1990, with significant reductions projected into the future. This indicates improvements in both early detection and treatment options, leading to fewer fatalities over time.

Similarly, Figure 6C shows that the ASR for DALYs related to POP followed an increasing trend until approximately 2010, after which a decline is projected through to 2040. This aligns with the observed trends in incidence and deaths, suggesting that the overall burden of the disease, in terms of disability, is expected to decrease as healthcare interventions improve.

## Discussion

The study investigates the spatiotemporal trends of POP that affects millions of women worldwide (11). The Study provides comprehensive data on POP’s impact across 195 countries. The incidence of POP increased notably from 1990–2021, while ASIR decreased. Similarly, the number deaths and DALYS due to POP increased from 1990–2021, however, the ASDR remained stable or slightly decreased. Thus, the EAPC of incidence, death and DALYs reduced. The ASRs for the incidence, deaths, and DALYs of POPs were highest in the low-SDI regions with a decreasing trend in all regions. A similar trend was also observed in previously reported studies (17–19). Regionally, highest ASIR, ASDR and DALYs were noted in South Asia, Southeast Asia, and East Asia. These high values can be attributed to the following reasons. These regions have larger family sizes. The larger family sizes and high population density can contribute to the spread of infectious diseases, increasing overall disease burden. Limited access to healthcare in some areas leads to delayed diagnosis and treatment, worsening disease outcomes. Moreover, early marriage and high childbirth rates increase reproductive strain, with frequent pregnancies limiting recovery time for pelvic muscles. Prolonged labor and unassisted deliveries, often due to limited maternal healthcare access, further weakens the pelvic support structures (12). These regions have high levels of air pollution, which is a major contributor to respiratory diseases. Industrial emissions, vehicle exhaust, and household air pollution from biomass fuel use are common. Tobacco consumption is prevalent in many parts of South and East Asia, increasing the risk of respiratory and cardiovascular diseases (20). Certain genetic markers may make populations in these regions more susceptible to specific diseases, including respiratory conditions. Moreover, disparities in social and economic factors influencing health are prevalent both within individual countries and across the broader region. Those most affected by these inequalities include individuals with limited education, marginalized communities, women, children, and the older adults, particularly those residing in rural areas and urban slums. These vulnerable populations bear a disproportionately high burden of disease, exacerbated by restricted access to healthcare, economic hardships, and social exclusion (12, 20, 21).

Preventing and managing POP in these countries requires region-specific strategies. In South and Southeast Asia, common risk factors include early marriage, high childbirth rates, and prolonged labor often linked to cultural norms and limited maternal healthcare. Education and awareness on pregnancy spacing, antenatal care, and skilled birth attendance can reduce childbirth-related trauma leading to POP. In East Asia, where aging populations are growing, pelvic floor exercises like Kegels help postmenopausal and postpartum women prevent POP progression (22, 23). Management strategies vary by region: in rural areas, vaginal pessaries offer low-cost symptom relief, while training healthcare workers in pessary fitting and post-op care is crucial. In urban settings, surgical interventions like pelvic floor repair are available but often hindered by financial barriers and social stigma. Additionally, culturally sensitive health programs promote reduced heavy lifting and pelvic health education further aid prevention. Tailoring interventions to each region’s socioeconomic, cultural, and healthcare challenges is key to reducing the burden of POP (22, 23).

Moreover, notable differences were also observed in their trends and high-income regions showed a remarkable decline in deaths and DALYs. In the meantime, various studies indicate that in low- and middle-income nations, the average prevalence of POP is 19.7%, with an estimated range of 3.4 to 56.4% (24). This data is consistent with our findings and suggests higher income and improved education facilities contribute to better health outcomes (25). The reason for better health outcomes is due to the reason that higher income and improved education can enhance healthcare access and overall health through multiple pathways. For instance, increased income allows better access to medical services, preventive care, nutrition, sanitation, and clean water, reducing disease burdens. Education promotes health literacy, leading to informed decisions on hygiene, nutrition, vaccination, and early illness detection. Maternal education improves childcare practices, reducing infant mortality and malnutrition. At a broader level, economic growth strengthens public health infrastructure, while education fosters health awareness. Together, they create a cycle of better healthcare access, disease prevention, and healthier lives (26, 27).

Evaluating the prevalence of POP is challenging due to the wide variations in definitions and diagnostic criteria found in both academic and clinical settings (14). Estimates indicate that the global prevalence might reach as much as 15% when based on symptomatic diagnosis and may soar to 64.6% using clinical evaluation (4, 5, 14). Bangladesh, Nigeria, the United States, and India have the highest age-specific incidence rates of this condition nationally. Data shows that in Uganda, the prevalence stands at 27.5% (28), while it’s 8% in Nepal (29), 10.3% in Pakistan (12), 15.6% in Bangladesh (30), and 9.6% in China (31). Symptomatic POP has the highest prevalence, reaching 18.6 per 1,000 individuals. Clearly, the onset of POP significantly impacts the quality of life (QoL) and impairs both social and personal activities. Overall, women with prolapse generally experience a lower QoL compared to the age-standardized population (32). While many patients felt their condition improved following non-surgical or surgical interventions, POP remains prevalent and untreated among women in rural areas (33). In summary, as a key factor influencing the health quality of older women, the rising incidence of POP is gaining increasing attention, making it essential to implement effective prevention and control measures to genuinely enhance the health outcomes for these women.

Moreover, the current study highlights the link between age and POP, showing how age affects its prevalence and impact. Notably, incidence and DALY rates begin to rise from the 25–29 age group and surge after age 40. This indicates that early intervention might be essential in middle age to lessen POP’s later effects. Previous studies also indicate that POP is highly prevalent among women over the age of 40, particularly in older adults and postmenopausal populations, with estimates suggesting a prevalence rate ranging from 41 to 50% (34, 35). The highest incidence rates in the 65–69 age group suggest a key point for implementing preventive measures in the older adults. The rising DALY rates, peaking in the 70–74 age group, reveal that increased incidence correlates with greater disability and diminished quality of life for older people. Approximately 11% of American women have undergone surgery for POP or urinary incontinence before reaching the age of 79, with an additional 29.2% potentially requiring further surgical intervention (36). It is important to highlight that high parity, obesity, and vaginal delivery are significant risk factors associated with POP (37). As women age, these factors can interact and exacerbate one another, making aging a critical contributor to the risk of developing POP (38). This emphasizes the urgent need for age-focused health policies, as the risks of disability and death linked to POP rise in older populations. Tailored public health efforts can create awareness and promote preventive care at crucial ages to lessen POP’s impact on individuals and health systems.

A comprehensive approach to POP prevention and management should target high-prevalence regions with tailored interventions for different age groups, engaging women, healthcare providers, and facilities in coordinated efforts. For adolescents and young women (15–25 years), education on pelvic floor health through schools and community programs can help prevent early risk factors. Awareness campaigns should discourage early marriages and pregnancies, which contribute to POP, while promoting healthy posture and physical activity to strengthen pelvic muscles. Among women of reproductive age (25–40 years), the focus should be on pregnancy care and childbirth safety. Encouraging antenatal care, pelvic floor exercises, and proper birth spacing can minimize pelvic trauma. Healthcare professionals should advocate for institutional deliveries to prevent complications from prolonged labor or repeated unassisted births. Additionally, educating women on avoiding heavy lifting and straining is crucial. For perimenopausal and postmenopausal women (40 + years), routine gynecological checkups can aid in early detection, while lifestyle modifications such as weight management and proper nutrition help maintain pelvic health. Supervised pelvic floor exercises can further reduce the progression of POP. Doctors play a vital role in screening, counseling, and providing non-surgical management, while gynecologists offer specialized care, including pessaries and minimally invasive treatments. Community health workers can support rural populations by promoting awareness and encouraging institutional deliveries. Healthcare facilities should implement community-based education programs, routine screenings, pelvic health rehabilitation clinics, and advanced treatment centers to ensure accessibility. Training healthcare workers in POP prevention and management, along with subsidizing surgical treatments for low-income groups, can enhance care options. Finally, community engagement through advocacy campaigns, government involvement, and media outreach can foster a preventative culture, reducing the burden of POP and ensuring equitable healthcare access. By integrating education, medical support, and policy interventions, this approach can significantly improve pelvic health outcomes.

### Limitations

This study has some unavoidable limitations that must be acknowledged. First, while the GBD provides valuable data on population health, discrepancies in data collection, coding, and quality across sources may influence the accuracy of the findings. Second, variations in incidence rates and annualized DALYs could reflect actual age-related trends but may also be affected by regional disparities, potentially introducing bias into the analysis. Improving population-based registries will be crucial for obtaining more precise estimates. Additionally, countries with lower socio-economic status often have limited health data, impacting the ability to assess the true burden of conditions such as POP. Underreporting is a significant concern, especially in low-income settings where healthcare access and data collection systems remain inadequate. This could result in conservative estimates of POP incidence, mortality, and DALYs, particularly in low-SDI regions where the highest age-standardized rates were recorded. Due to diagnostic challenges and incomplete data, the actual burden may be higher than reported, affecting conclusions about regional disparities. Moreover, improving data collection through community-based surveys and mobile health initiatives will be essential to obtaining more accurate estimates. Despite these limitations, this study provides the most up-to-date global estimates of POP, offering valuable insights for public health policymakers to develop effective strategies for addressing the issue and its associated risk factors.

## Conclusion

Based on GBD 2021 data, this study analyzed the global burden of POP from 1990 to 2021. The findings reveal both similarities and differences in POP burden across SDI regions, offering insights for future health strategies. There are significant global and regional disparities in POP burden by age. High SDI regions have reduced POP incidence and prevalence, while low-middle and low SDI areas face increasing challenges. This highlights the urgent need for targeted public health efforts and resource allocation in low SDI regions. To effectively prevent and manage POP, a comprehensive program must focus on high-prevalence regions and key age groups while ensuring coordinated efforts from women, healthcare providers, and medical institutions. Such a program should integrate pelvic-floor health education into school, college, and community health programs, fostering early awareness. It should also address socio-cultural factors by discouraging early child marriages, promoting family planning, and advocating for safe childbirth practices. Workshops can equip women with knowledge on minimizing physical stress on the pelvic floor, preventing prolonged labor, and avoiding unsafe home deliveries or repeated unassisted vaginal births. Additionally, healthcare providers should receive training on early detection and non-surgical management options, ensuring accessible care for underserved populations. A multi-faceted approach combining education, medical support, and policy interventions is essential for effectively reducing the burden of POP.

## Data Availability

The original contributions presented in the study are included in the article/supplementary material, further inquiries can be directed to the corresponding authors.
